# Associations of the Lifestyle for Brain Health Index With Structural Brain Changes and Cognition: Results From the Maastricht Study

**DOI:** 10.1212/WNL.0000000000012572

**Published:** 2021-09-28

**Authors:** Irene S. Heger, Kay Deckers, Miranda T. Schram, Coen D.A. Stehouwer, Pieter C. Dagnelie, Carla J.H. van der Kallen, Annemarie Koster, Simone J.P.M. Eussen, Jacobus F.A. Jansen, Frans R.J. Verhey, Martin P.J. van Boxtel, Sebastian Köhler

**Affiliations:** From the School for Mental Health and Neuroscience (I.S.H., K.D., M.T.S., J.F.A.J., F.R.J.V., M.P.J.v.B., S.K.), Department of Psychiatry and Neuropsychology (I.S.H., K.D., F.R.J.V., M.P.J.v.B., S.K.), School for Cardiovascular Diseases (M.T.S., C.D.A.S., P.C.D., C.J.H.v.d.K., S.J.P.M.E.), Care and Public Health Research Institute (A.K.), Department of Social Medicine (A.K.), and Department of Epidemiology (S.J.P.M.E.), Maastricht University; and Heart and Vascular Center (M.T.S.), Department of Internal Medicine (M.T.S., C.D.A.S., P.C.D., C.J.H.v.d.K.), and Department of Radiology (J.F.A.J.), Maastricht University Medical Center+, the Netherlands.

## Abstract

**Background and Objectives:**

Observational research has shown that a substantial proportion of all dementia cases worldwide are attributable to modifiable risk factors. Dementia risk scores might be useful to identify high-risk individuals and monitor treatment adherence. The objective of this study was to investigate whether a dementia risk score, the Lifestyle for Brain Health (LIBRA) index, is associated with MRI markers and cognitive functioning/impairment in the general population.

**Methods:**

Cross-sectional data were used from the observational population-based cohort of The Maastricht Study. The weighted compound score of LIBRA (including 12 dementia risk and protective factors, e.g., hypertension, physical inactivity) was calculated, with higher scores indicating higher dementia risk. Standardized volumes of white matter, gray matter, and CSF (as proxy for general brain atrophy), white matter hyperintensities, and presence of cerebral small vessel disease were derived from 3T MRI. Cognitive functioning was tested in 3 domains: memory, information processing speed, and executive function and attention. Values ≤1.5 SDs below the average were defined as cognitive impairment. Multiple regression analyses and structural equation modeling were used, adjusted for age, sex, education, intracranial volume, and type 2 diabetes.

**Results:**

Participants (n = 4,164; mean age 59 years; 49.7% men) with higher LIBRA scores (mean 1.19, range −2.7 to 9.2), denoting higher dementia risk, had higher volumes of white matter hyperintensities (β = 0.051, *p* = 0.002) and lower scores on information processing speed (β = −0.067, *p* = 0.001) and executive function and attention (β = −0.065, *p* = 0.004). Only in men, associations between LIBRA score and volumes of gray matter (β = −0.093, *p* < 0.001) and CSF (β = 0.104, *p* < 0.001) and memory (β = −0.054, *p* = 0.026) were found. White matter hyperintensities and CSF volume partly mediated the association between LIBRA score and cognition.

**Discussion:**

Higher health- and lifestyle-based dementia risk is associated with markers of general brain atrophy, cerebrovascular pathology, and worse cognition, suggesting that LIBRA meaningfully summarizes individual lifestyle-related brain health. Improving LIBRA factors on an individual level might improve population brain health. Sex differences in lifestyle-related pathology and cognition need to be further explored.

**Classification of Evidence:**

This study provides Class II evidence that higher LIBRA scores are significantly associated with lower scores in some cognitive domains and a higher risk of cognitive impairment.

A substantial proportion of dementia cases might be attributable to modifiable risk factors.^1,2^ Early detection of individuals at risk, allowing timely management, has great public health implications,^1^ as echoed by recent reports of the *Lancet* Commission on Dementia Prevention, Intervention and Care^2^ and the World Health Organization (WHO).^3^

Dementia risk scores, summarizing individual risks, might be useful for the selection of high-risk individuals and could serve as intermediate outcomes to monitor treatment adherence. Some risk scores have been associated with structural brain changes and cognitive functioning,^4-7^ but most are based on single cohort studies or include factors that are not amenable to change, e.g., age,^4-8^ known to be highly correlated with brain markers. The Lifestyle for Brain Health (LIBRA) index is based on a systematic literature review and Delphi consensus on factors amendable to change,^9^ thereby summarizing one's potential for brain health improvement.^9^ Criterion validity has been established by several prospective studies relating higher LIBRA scores to steeper cognitive decline, incident cognitive impairment, and dementia in midlife and late life,^9-14^ as well as intervention effects in multifactorial randomized controlled trials.^15^ Whether LIBRA score is also related to brain markers, reflecting more direct neurobiological markers of brain health, remains to be elucidated.

Therefore, this study aimed to examine the association of LIBRA score with cognitive performance and impairment and evidence of neuroimaging abnormalities in the general adult population (age 40–75 years). In addition, we investigated biological plausible pathways by testing whether MRI markers mediated the association of LIBRA score with cognition.

## Methods

### Participants

Data were used from The Maastricht Study, an observational population-based cohort study, the rationale and methodology of which have been described previously.^16^ In brief, the study focuses on the etiology, pathophysiology, complications, and comorbid conditions of type 2 diabetes (T2D) and is characterized by an extensive phenotyping approach. Individuals between 40 and 75 years of age and living in the southern part of the Netherlands were eligible for participation. Participants were recruited through mass media campaigns and from the municipal registries and the regional Diabetes Patient Registry (which includes virtually all individuals with T2D in primary, secondary, or tertiary care in the targeted population) via mailings. Recruitment was stratified according to known T2D status, with an oversampling of individuals with T2D, for reasons of efficiency, while at the same time monitoring the representation of the source population continuously.^16,17^ The present report addresses several primary research questions. Are higher (i.e., more unhealthy) LIBRA scores associated with lower scores on cognitive functioning and a higher odds of cognitive impairment (Class II evidence)? Are higher LIBRA scores associated with lower volumes of MRI markers and a higher odds of cerebral small vessel disease (CSVD) (Class II evidence)? To what extent can volumetric MRI markers explain the association between LIBRA and cognitive functioning (Class II evidence)? Cross-sectional data were used from participants who completed the baseline survey between November 2010 and January 2018. The examinations of each participant were performed within a time window of 3 months. MRI measurements were implemented from December 2013 onward. Participants were included in the analyses if data on MRI outcomes, at least 11 LIBRA factors (Table 1), and cognition were available.

**Table 1:**
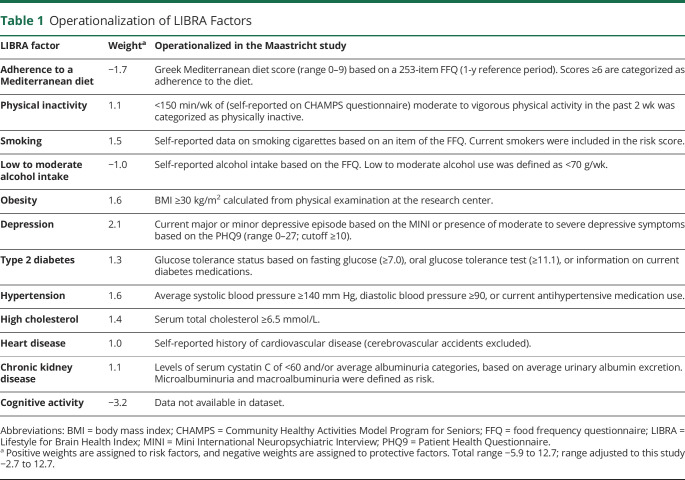
Operationalization of LIBRA Factors

### Operationalization of the LIBRA Score

The individual LIBRA factors were created on the basis of clinical data from physical examination or self-reported questionnaires from the baseline measurement of The Maastricht Study and then dichotomized (presence of LIBRA factor yes/no) according to established cutoffs. The LIBRA total score is computed by assigning a weight (positive for presence of risk factors; negative for presence of protective factors) to each factor according to the relative risks from published meta-analyses.^9,18^ Weights are then standardized and summed to a total score. A higher LIBRA score reflects higher dementia risk, with scores ranging from −5.9 to 12.7.^9^ All LIBRA factors could be operationalized in The Maastricht Study except for the LIBRA factor high cognitive activity. Engagement in cognitively stimulating activities was not available in the dataset; therefore, this LIBRA factor could not be included in the risk calculation. Available protective factors were adherence to a Mediterranean diet and low to moderate alcohol use. Risk factors were physical inactivity, smoking, obesity, depression, T2D, hypertension, hypercholesterolemia, heart disease, and chronic kidney disease. Table 1 provides an overview of all individual LIBRA factors, assigned weights, and operationalization in this dataset.

Adherence to a Mediterranean diet was based on the Greek Mediterranean diet score derived from a comprehensive 253-item self-administered food frequency questionnaire (FFQ) on frequency (not used to used 7 d/wk) and consumed amounts (<1–>12 per day), with a 1-year reference period.^19^ The Mediterranean diet score consists of the reported intake of vegetables, fruit and nuts, fish, cereal intake, dairy, meat, and alcohol, with scores ranging from 0 to 9. A score of ≥6 is used as a cutoff for adhering to the diet.^20^ Nonadherence to this diet does not necessarily imply nonadherence to the Dutch food-based dietary guidelines, which provide a more general guideline for a healthy diet in relation to numerous chronic diseases than specifically for brain health and dementia.^21^ Physical inactivity was based on self-reported moderate to vigorous physical activity in the past 2 weeks, calculated from a modified version of the Community Healthy Activities Model Program for Seniors questionnaire.^22^ Less than 150 min/wk of moderate to vigorous physical activity was categorized as physically inactive, according to the Dutch physical activity guidelines.^23^ Smoking status was defined by self-reported data on smoking cigarettes, with response options of never smoked, ever smoked, and currently smoking. Current smokers were assigned to the risk group. Low to moderate alcohol use was based on self-reported alcohol use per day based on an item of the FFQ, converted into grams of ethanol per day. Low to moderate alcohol intake was defined as ≤70 g/wk, based on the Dutch guidelines recommending not to drink or to drink no more than 1 glass of alcohol a day.^21^ Obesity was based on the WHO categories,^24^ in which a body mass index (calculated from physical examination at the research center) of ≥30 kg/m^2^ was defined as obese. The presence of depression was assessed with the Mini International Neuropsychiatric Interview (current major or minor depressive episode).^25^ In case of missing data on the Mini International Neuropsychiatric Interview, the Patient Health Questionnaire was used to determine presence of moderate to severe depressive symptoms (range 0–27; cutoff ≥10).^26^ T2D was defined according to glucose tolerance status based on fasting glucose (≥7.0) or oral glucose tolerance test (≥11.1), according to the WHO definition, or based on information on current diabetes medication use.^27^ For sensitivity analyses, a second variable was computed that was based on impaired glucose metabolism, which includes both prediabetes and T2D. Hypertension was based on average office blood pressure measurement (systolic blood pressure ≥140 mm Hg or diastolic blood pressure ≥90 mm Hg) or current antihypertensive medication use. Hypercholesterolemia was calculated from serum total cholesterol using a cutoff of ≥6.5 mmol/L. The LIBRA factor heart disease was based on self-reported history of cardiovascular disease from the Rose Questionnaire^28^ (i.e., myocardial infarction, and/or percutaneous artery angioplasty of the coronary arteries, abdominal arteries peripheral arteries or carotid artery, and/or vascular surgery on coronary arteries, abdominal arteries peripheral arteries, or carotid artery). Presence of cerebrovascular infarction and presence of hemorrhage were not included in the risk calculation of the LIBRA factor heart disease. For sensitivity analyses, a second variable was computed that was based only on self-reported history of myocardial infarction,^29^ thereby including only coronary heart disease. Chronic kidney disease was derived from *Chronic Kidney Disease* Epidemiology Collaboration equation–estimated glomerular filtration rate using serum cystatin C (serum cystatin C of <60) or average urinary albumin excretion (both microalbuminuria and macroalbuminuria defined as risk).^30^

### Cognitive Performance

Cognitive performance was assessed by a concise (30-minute) neuropsychological test battery.^16^ For conceptual clarity, individual neuropsychological test scores were standardized and divided into 3 cognitive domains (memory function, information processing speed, and executive function and attention (reprinted with permission).^16,17^ Briefly, memory function was evaluated with the Verbal Leaning Test,^31^ and a memory domain score was derived by calculating the average of total immediate and delayed recall standardized scores. An information processing speed domain score was derived from standardized scores of the Stroop Color-Word Test parts I and II,^32^ the Concept Shifting Test parts A and B,^33^ and the Letter-Digit Substitution Test.^34^ The executive function and attention domain score was calculated from the average score of the Stroop Color-Word Test part III and the Concept Shifting Test part C. If necessary, individual test scores were log-transformed to reduce skewness of distributions or inverted so that higher scores indicated better cognitive performance. In addition, participants were categorized as cognitively impaired (yes/no) on the basis of a regression-based normalization procedure per test that predicted expected scores for each individual given their age, sex, and level of education from a published normative sample.^31-34^ The difference between observed and expected scores and their SDs were used to calculate *z* scores, which were then averaged per domain and restandardized. Individuals performing ≤1.5 SDs below their norm-based expected score in any of the 3 cognitive domains were categorized as having cognitive impairment.

### Brain MRI

Brain MRI was performed on a 3T MRI scanner (MAGNETOM Prismafit Syngo MR D13D; Siemens Healthcare, Erlangen, Germany) with the use of a 64-element head coil for parallel imaging, as previously described.^16^

### Measurement of Brain Volumes and Cerebral Small Vessel Disease

T1 images and T2-weighted fluid-attenuated inversion recovery images were analyzed by use of an ISO-13485:2012 certified automated method (which included visual inspection).^35,36^ T1 images were segmented into gray matter (GM), white matter (WM), and (as an inverted measure of brain atrophy) CSF (1 voxel = 1.00 mm^3^ = 0.001 mL).^35^ Intracranial volume was calculated as the sum of GM, WM, and CSF. T2-weighted fluid-attenuated inversion recovery and T1 images were used to calculate WM hyperintensity (WMH) volume.^36^ Identified WMHs were summed to assess total WMH burden in milliliters. In addition, WMHs were visually rated with the Fazekas scale.^37^ Lacunar infarcts and cerebral microbleeds were counted manually by 3 neuroradiologists in accordance with the Microbleed Anatomical Rating Scale.^38,39^ Presence of CSVD was defined as a Fazekas score of ≥2, presence of lacunar infarcts, or presence of cerebral microbleeds.

### Statistical Analysis

Independent-samples *t* tests and χ^2^ tests were used to investigate differences in demographic variables and LIBRA scores between the actual study sample used in the present study and the excluded group and between 3 LIBRA score groups (low risk: ≤1 SD below sample mean; middle risk: between −1 and 1 SD; and high risk: ≥1 SD above sample mean). The associations between LIBRA and the structural MRI markers and between LIBRA and the 3 cognitive domains were analyzed in separate multiple linear regression analyses. A quadratic term of LIBRA was added to the linear function in the analyses of the cognitive domains information processing speed and executive function and attention because this improved model fit. For direct comparison of strength of associations, we report the standardized regression coefficient β and 95% confidence interval (CI). Logistic regression analyses were used to examine the association between LIBRA score and CSVD and between LIBRA score and cognitive impairment, yielding odds ratios (ORs) and 95% CIs.

**Figure 1:**
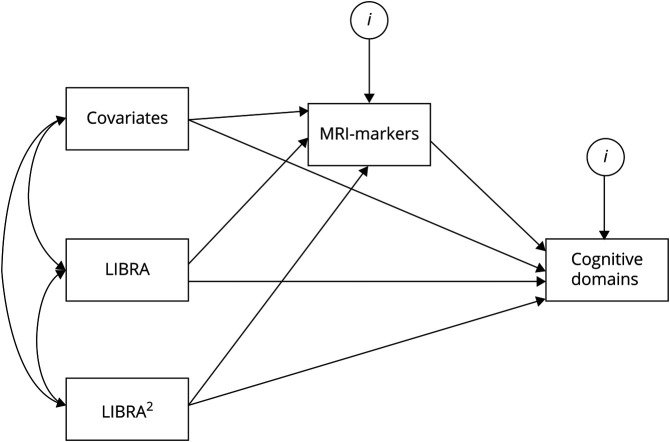
Path Model to Quantify the Instantaneous Indirect Effect of LIBRA Score on Cognition Covariates: sex, age, level of education, time between assessment and MRI, intracranial volume (ICV), and diabetes status. LIBRA = Lifestyle for Brain Health (continuous); LIBRA^2^ = LIBRA squared. ^i^Standard error.

Structural equation modeling was used to study mediation of LIBRA score on cognition by MRI markers by decomposing the total association into direct and indirect associations. Because the regression analysis suggested a curvilinear association between LIBRA score and 2 cognitive domains, we used a technique that allows estimating of nonlinear mediation effects, which is not taken into account in traditional linear or log-linear mediation models (Figure 1).^40^ For this, we estimated the instantaneous indirect effect θ, which tests the mediation effect at different levels of the independent predictor variable (LIBRA score), showing how the mediation effects change as the level of the independent variable changes. Following this approach, we estimated the instantaneous indirect effects θ at 3 levels of LIBRA score: 1 SD below the LIBRA sample mean (LIBRA score −0.87), at the LIBRA sample mean (LIBRA score 1.19), and 1 SD above the LIBRA sample mean (LIBRA score 3.25), following previous recommendations.^40^ To estimate robust 95% CIs, we used bootstrapping with 10,000 repetitions.

Associations with cognition were adjusted for age, sex, and level of education. Associations with structural brain markers were in addition adjusted for intracranial volume to correct for head size and the variable MRI lag time to adjust for the time (in years) between inclusion and MRI scan. The oversampling of participants with T2D by design urged us to adjust for diabetes status in all the analyses to ensure that the overexpression of LIBRA risk factors in T2D such as obesity, hypercholesterolemia, hypertension, or depression did not confound the observed associations between LIBRA score, MRI markers, and cognition. Interaction terms were included in additional analyses to investigate whether the associations between LIBRA scores and brain markers or cognitive performance were moderated by sex and T2D status. Finally, we did a series of sensitivity analyses to test the robustness of findings after assigning those with prediabetes the risk weight for T2D and after assigning a risk weight only to those with coronary heart disease. Statistical analyses were done with Stata 13.1 (StataCorp, College Station, TX) and Mplus8 (Muthen & Muthen) using 2-sided hypothesis testing and an α level of <0.05.

### Standard Protocol Approvals, Registrations, and Patient Consents

The Maastricht Study has been approved by the institutional medical ethical committee (NL31329.068.10) and the Ministry of Health, Welfare and Sports of the Netherlands (permit 131088-105234-PG). All participants gave their written informed consent.^16^

### Data Availability

Data are unsuitable for public deposition due to ethical restrictions and privacy regulation of participant data. Data from The Maastricht Study are available to any interested researcher who meets the criteria for access to confidential data. Data requests may be submitted to The Maastricht Study Management Team.

## Results

### Study Design and Sample Characteristics

**Figure 2:**
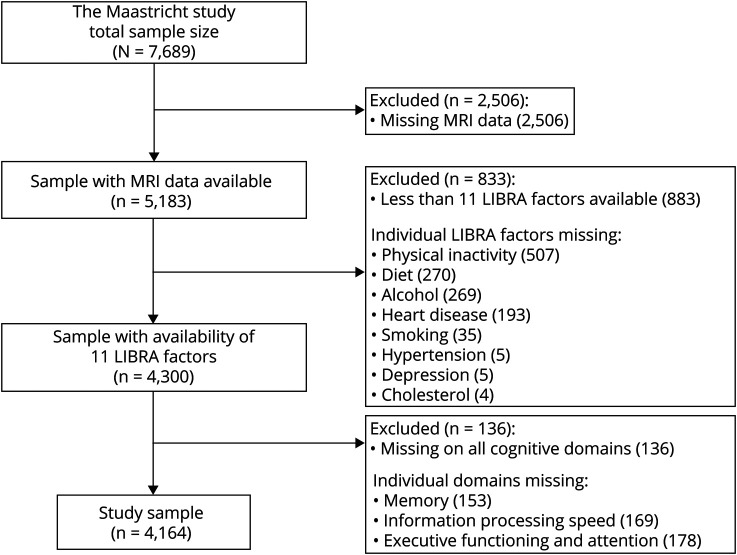
Flowchart of the Study Sample Selection LIBRA = Lifestyle for Brain Health.

Of all 7,689 participants (mean age 59.8 years; 50.4% men; 34.7% low educated; 24.6% T2D), 45.8% were excluded from the present study, largely due to absence of MRI data. LIBRA factors that were most often missing were physical inactivity (9.8% missing) and adherence to a Mediterranean diet and low to moderate alcohol intake (from the same food questionnaire; 5.2% missing). All other LIBRA factors were <3.7% missing. Figure 2 provides a flowchart. Compared to the study sample (n = 4,164), excluded participants (n = 3,525) had a higher mean age (59.2 years vs 60.5 years; *t*[7,687] = 6.5, *p* < 0.001) and had lower education (sample low education 30.2%, excluded low education 40.2%; χ^2^[2] = 86.6, *p* < 0.001). Excluded participants had a more unfavorable LIBRA risk profile (1.19 vs 1.95; *t*[7,687] = 15.4, *p* < 0.001), with a higher presence of T2D (19.0% vs 31.3%; χ^2^[1] = 156.1, *p* < 0.001), hypertension (49.0% vs 59.7%; χ^2^[1] = 87.0, *p* < 0.001), heart disease (10.1% vs 20.3%; χ^2^[1] = 152.3, *p* < 0.001), obesity (18.0% vs 25.9%; χ^2^[1] = 70.9, *p* < 0.001), chronic kidney disease (5.2% vs 7.6%; χ^2^[1] = 19.3, *p* < 0.001), and depression (4.2% vs 6.1%; χ^2^[1] = 13.7, *p* < 0.001). They were more often smokers (11.0% vs 16.4%; χ^2^[1] = 47.5, *p* < 0.001) and physically inactive (25.3% vs 31.6%, χ^2^[1] = 31.9, *p* < 0.001) and less often adhered to the Mediterranean diet (28.5% vs 26.2%; χ^2^[1] = 4.6, *p* = 0.032). Low to moderate alcohol intake was more common in the excluded group (54.9% vs 59.4%; χ^2^[1] = 14.7, *p* < 0.001), and hypercholesterolemia was more common in the study sample compared to excluded participants (15.4% vs 12.3%; χ^2^[1] = 14.4, *p* < 0.001). Men had higher (unhealthier) average LIBRA scores (1.5) compared to women (0.9; *t*[4,162] = 10.3, *p* < 0.001), including higher presence of T2D (25.6% vs 12.5%, χ^2^[1] = 116.1, *p* < 0.001), hypertension (57.7% vs 40.4%, χ^2^[1] = 125.1, *p* < 0.001), and physical inactivity (28.1% vs 22.5%, χ^2^[1] = 17.1, *p* < 0.001). The characteristics of the total study sample and those with a low (≤1 SD below sample mean), middle (between −1 and 1 SD), and high (≥1 SD above sample mean) LIBRA score are summarized in Table 2.

**Table 2:**
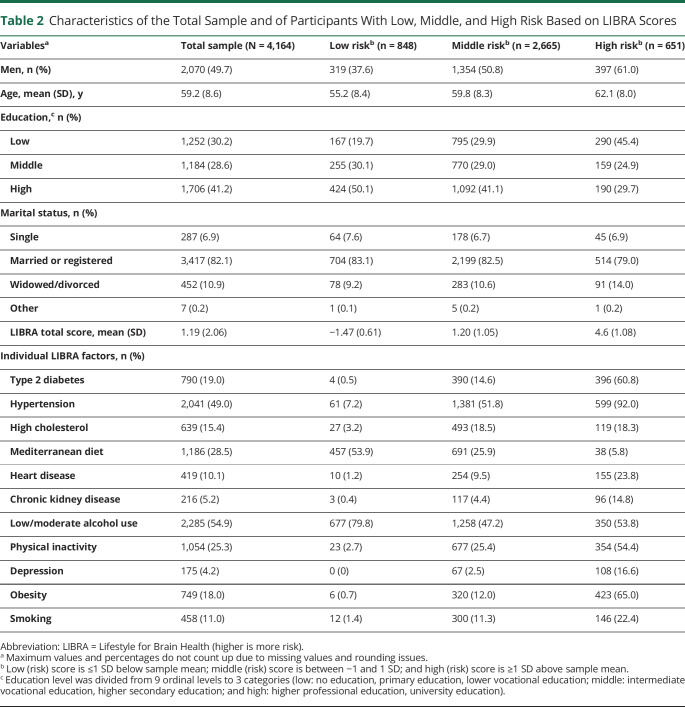
Characteristics of the Total Sample and of Participants With Low, Middle, and High Risk Based on LIBRA Scores

### LIBRA Score and Structural Brain Measures

**Figure 3:**
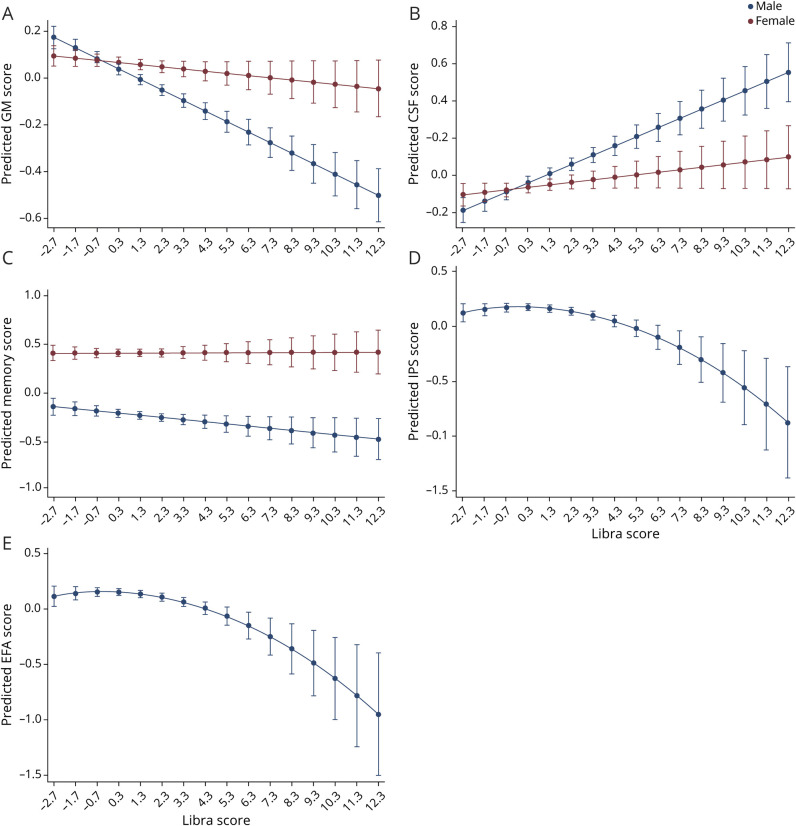
LIBRA Score and MRI and Cognition: Differences in Sex Estimated marginal means showing the (sex-specific) associations between Lifestyle for Brain Health (LIBRA) score and (A) gray matter (GM) volume, (B) CSF volume, (C) memory function, (D) information processing speed (IPS), and (E) executive function and attention (EFA).

Table 3 displays the results of the multiple linear regression analyses of the association between LIBRA score and the volumetric MRI markers. Higher LIBRA scores were linearly associated with higher volumes of WMH in the total sample. Interaction analyses revealed that the associations between LIBRA score and GM and CSF volumes were moderated by sex, with stronger and significant associations in men, but associations in women were directionally similar (Figure 3, A and B). No association was found between LIBRA score and volume of WM, and no interactions were found by T2D status. There was no association between the LIBRA score and presence of CSVD (OR 1.036, 95% CI 0.994–1.080, *p* = 0.092). When a stricter definition of CSVD, defined as the presence of at least 2 markers of CSVD, was applied, an association was found (OR 1.123, 95% CI 1.028–1.226, *p* = 0.010).

**Table 3:**
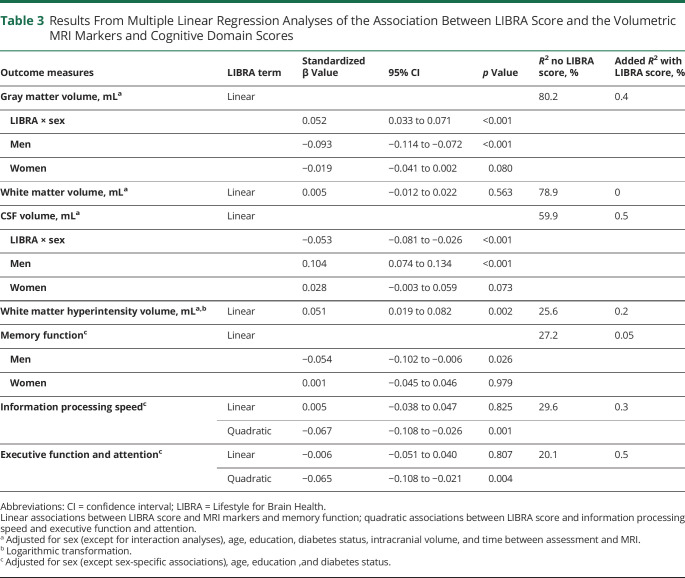
Results From Multiple Linear Regression Analyses of the Association Between LIBRA Score and the Volumetric MRI Markers and Cognitive Domain Scores

### LIBRA Score and Cognition

Likelihood ratio testing of the association between LIBRA score and cognition showed that the model including both a linear and a quadratic LIBRA term had the best fit for the cognitive domains information processing speed and executive function and attention. As Figure 3, C–E shows, the relationship between LIBRA score and these 2 domains changed as LIBRA scores increased in a curvilinear fashion, with a stronger negative association as LIBRA scores increased. A linear LIBRA term was the best fit for the domain of memory function. The results of the regression analyses are displayed in table 3. Wald tests of the joint effects of the combined linear-quadratic LIBRA term were significant for both information processing speed (*F*_2, 4,099_ = 9.08, *p* < 0.001) and executive function and attention (*F*_2, 4,090_ = 9.14, *p* < 0.001). In addition, we filtered the model only to cognition scores > −1 and then performed Wald tests to test whether the quadratic LIBRA score still improved the model. Wald tests were significant for both information processing speed (*p* < 0.001) and executive function and attention (*p* = 0.007). No interactions were found for sex and T2D status. Sex-specific analyses suggested that the effect for memory function was present only in men (Figure 3C and Table 3).

### Cognitive Impairment

Likelihood ratio testing showed that the model including both a linear and a quadratic LIBRA term had the best fit for cognitive impairment. Logistic regression analyses revealed a relationship between the quadratic LIBRA score and the odds of cognitive impairment (OR 1.02, 95% CI 1.006–1.036, *p* = 0.006).

### Mediation Analyses of the Association Between LIBRA Score and Cognitive Outcomes by MRI Markers

Nonlinear mediation at different levels of LIBRA score (low: −1 SD; middle: at mean; high: 1 SD) showed that WMH volumes partly explained the relationship between LIBRA score and information processing speed, executive function and attention, and cognitive impairment in the total sample. Following the observed curvilinear association between LIBRA and these cognitive outcomes, the nonlinear mediation effect θ tended to increase across levels of LIBRA score. This suggests that MRI markers partly mediated the association between LIBRA score and cognitive outcomes, and this became even stronger as LIBRA score increased. Higher CSF volumes also mediated the association between LIBRA score and information processing speed and between LIBRA score and executive function and attention. In men only, WMH volumes mediated the association between LIBRA score and memory function. Details on the estimations of the (instantaneous) indirect associations of LIBRA score on cognition through MRI are given in Table 4.

**Table 4:**
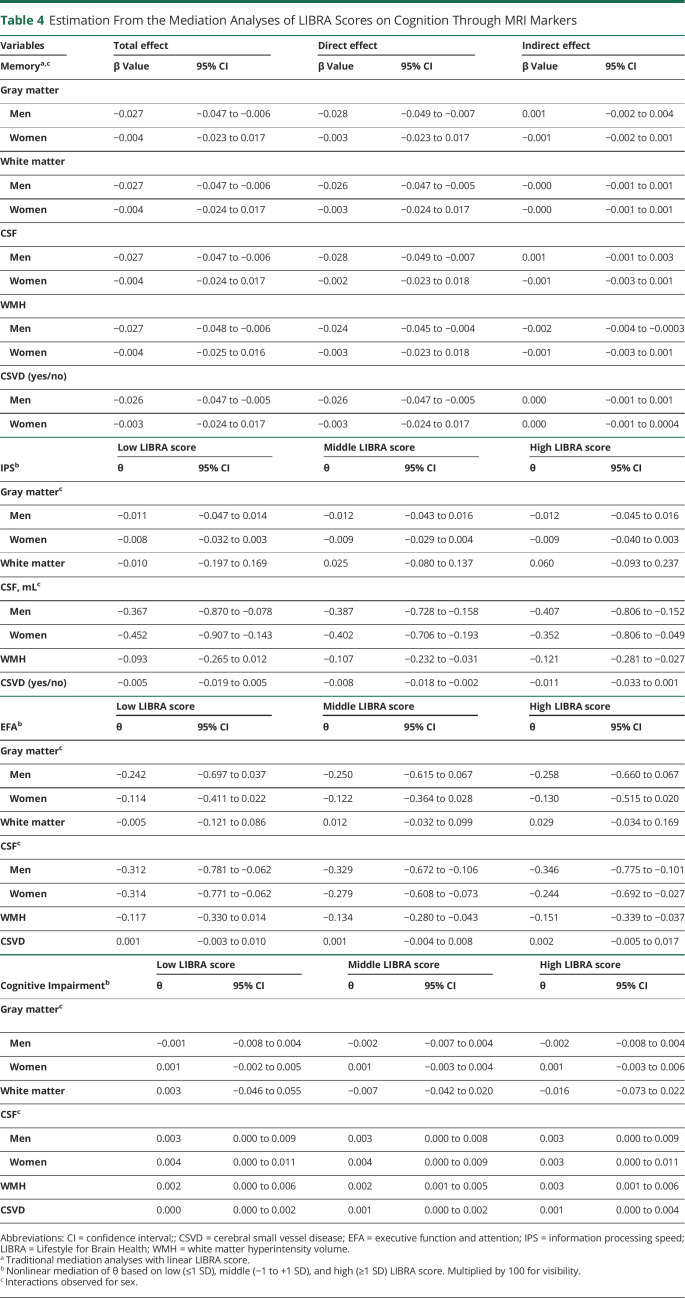
Estimation From the Mediation Analyses of LIBRA Scores on Cognition Through MRI Markers

### Additional Analyses

Sensitivity analyses were performed for the LIBRA variables T2D, assigning those with prediabetes the risk weight for T2D, and heart disease, by assigning a risk weight only to those with coronary heart disease (in line with its initial use in LIBRA^29^). Results remained similar to the main analyses.

## Discussion

This cross-sectional population-based study investigated the relationship of a modifiable risk score for dementia with brain MRI markers and cognitive functioning. Higher LIBRA scores, reflecting a less brain-healthy lifestyle, were associated with WMH volume, with lower scores on information processing speed and executive function and attention, and higher odds of cognitive impairment. Associations of LIBRA score with memory and general brain atrophy (i.e., GM, CSF) were present only in men. Volumes of WMH and CSF mediated the association between LIBRA score and cognition in the full cohort, and WMH mediated the relation with memory in men.

The results confirm previous studies showing that higher LIBRA scores are related to lower cognitive functioning and higher risk for cognitive impairment and dementia in the general population and clinical studies.^10-14^ Our study shows a relationship of LIBRA score with underlying biological gradients of WMH and global atrophy using population MRI, showing that it is indeed an index of brain health. In men, higher LIBRA scores were associated with higher volumes of brain atrophy and lower scores on memory function, with directionally similar but not significant associations in women. Although the association of LIBRA score with memory in men was found only in sex-specific analyses, not in formal interaction analyses as has been found for the association with brain atrophy, these 2 findings seem congruent. Both memory decline and brain atrophy are manifestations of Alzheimer disease,^41^ and previous studies showed that, in middle age, men have more pronounced brain atrophy compared to women, whereas women show steeper decline in later phases.^42-44^ Lifestyle-related brain damage might thus be more pronounced in men compared to women of the same age in our cohort who were 40 to 75 years of age, leading to lower cognitive performance. Indeed, men had higher mean LIBRA scores, which is in line with a previous study,^13^ as well as higher WMH and CSF volume and worse cognitive scores, including memory, than women in the present study. The fact that worse cognitive performance was more strongly related with MRI markers as LIBRA scores increased adds to the validity of this score for identifying those with low brain health and high risk of deterioration.

Various pathophysiologic mechanisms may affect the different LIBRA factors such as arteriolosclerosis,^45^ atherosclerotic burden,^46^ cerebral hypoperfusion,^46^ and neurodegenerative Alzheimer disease pathology.^47^ We found an association between LIBRA score and the presence of CSVD only when using a stricter definition of CSVD, which was not the initial a priori definition. Both WMH and brain atrophy explained the relation between LIBRA score and cognition. While cross-sectional associations do not allow temporal inference, it is in line with the idea that these risk factors affect and accelerate both vascular and neurodegenerative pathology.^48^ In line with our study, the Cardiovascular Risk Factors, Aging, and Incidence of Dementia (CAIDE) Risk Score also has been associated with WMH load.^49^ This score includes both modifiable (hypertension, hypercholesterolemia, body mass index, and physical inactivity) and nonmodifiable (e.g., age, sex, education) factors, which makes it difficult to disentangle their relative contribution. Besides, an external validation study of 4 dementia prediction models (including CAIDE) showed that age alone already showed nearly identical discriminative ability compared to the full model including other (modifiable) risk factors.^50^ We showed that a compound score of health and lifestyle factors is associated with brain markers and cognition, even after adjustment for the contribution of the nonmodifiable factors age, sex, and education. The use of a compound score that includes risk and protective factors all within the reach of vascular risk management and lifestyle interventions makes the LIBRA index a useful tool in identifying a group of individuals at increased risk of cognitive decline and dementia and for monitoring treatment targets over time in dementia prevention trials.^15^

Strengths of this study include the population-based design and the large sample size within the midlife target age range (40–75 years), which makes the results particularly generalizable to middle-aged individuals. Furthermore, the availability of domain-specific cognition and state-of-the-art population imaging data allowed us to study the association pathways in considerable depth. The extensive phenotyping approach of The Maastricht Study made it possible to operationalize the LIBRA factors largely on the basis of objective clinical data from physical examination at the research center, if applicable, in combination with self-reported data. Last, this study has taken the nonlinear association between LIBRA score and cognition into account in the mediation analysis by estimating the instantaneous indirect effects of the MRI markers. Unfortunately, these analyses made it impossible to quantify the overall indirect effect of LIBRA score on cognition through the MRI markers because the instantaneous indirect effect is estimated for 3 specific values of LIBRA (based on the SD).^40^ Other limitations of our study are the cross-sectional design, in which definitive conclusions concerning cause and effect are not possible. In addition, selection bias may have occurred in this present study due to missing MRI data. Indeed, the group who did not undergo an MRI and therefore were not included in this study were older, had a lower level of education, and appeared to be frailer, that is, had a higher presence LIBRA factors such as T2D, hypertension, and heart disease, which likely led to an underestimation of the associations. Next, while data on most LIBRA factors were available in this dataset, the absence of the LIBRA factor cognitive activity, which is the strongest protective factor (LIBRA weight of −3.2), could have weakened the predictive value of the LIBRA index. Furthermore, the use of dichotomous LIBRA scores, that is, presence of LIBRA factor yes/no, makes the index less suitable to detect small changes in a specific factor in behavioral change programs.^15^ Yet, a study showed that LIBRA was most responsive to change compared to other risk indices, probably due to the large number of modifiable factors.^15^ Still, the use of alternative scoring formats need to be considered. Finally, the adjustment for T2D status in all analyses might not be sufficient to control for the oversampling of participants with T2D by design. There was, however, no interaction pattern for T2D, suggesting that LIBRA scores had similar associations with cognition in those with and without T2D.

Future studies should replicate these findings in a prospective design to expand the understanding of the relationship between health- and lifestyle-related risk factors and cognitive aging over time. Furthermore, the mediation analyses should be explored further by more extensive brain structure measures (e.g., WM connectivity, hippocampal volume).

This study showed that higher LIBRA scores, indicating a less brain-healthy lifestyle profile, are associated with lower information processing speed, executive function and attention, and WMH in the total population and with lower memory function and markers of global brain atrophy in men, independently of the nonmodifiable risk factors age, sex, and education. Sex differences in the lifestyle-related pathology and manifestations of dementia need to be further explored. Improving health and lifestyle factors captured by LIBRA might improve population brain health.

## Glossary

CAIDE: Cardiovascular Risk Factors, Aging, and Incidence of Dementia
CI: confidence interval
CSVD: cerebral small vessel disease
FFQ: food frequency questionnaire
GM: gray matter
LIBRA: Lifestyle for Brain Health
OR: odds ratio
T2D: type 2 diabetes
WHO: World Health Organization
WM: white matter
WMH: WM hyperintensity

## References

[1] Norton S, Matthews FE, Barnes DE, Yaffe K, Brayne C. Potential for primary prevention of Alzheimer's disease: an analysis of population-based data. Lancet Neurol. 2014;13(8):788-794. 25030513 10.1016/S1474-4422(14)70136-X

[2] Livingston G, Huntley J, Sommerlad A, et al. Dementia prevention, intervention, and care: 2020 report of the *Lancet* Commission. Lancet. 2020;396(10248):413-446. 32738937 10.1016/S0140-6736(20)30367-6 PMC7392084

[3] Risk Reduction of Cognitive Decline and Dementia: WHO Guidelines. World Health Organization; 2019. 31219687

[4] Stephen R, Liu Y, Ngandu T, et al. Associations of CAIDE dementia risk score with MRI, PIB-PET measures, and cognition. J Alzheimers Dis. 2017;59(2):695-705. 28671114 10.3233/JAD-170092 PMC5523839

[5] Kivipelto M, Ngandu T, Laatikainen T, Winblad B, Soininen H, Tuomilehto J. Risk score for the prediction of dementia risk in 20 years among middle aged people: a longitudinal, population-based study. Lancet Neurol. 2006;5(9):735-741. 16914401 10.1016/S1474-4422(06)70537-3

[6] Reitz C, Tang MX, Schupf N, Manly JJ, Mayeux R, Luchsinger JA. A summary risk score for the prediction of Alzheimer disease in elderly persons. Arch Neurol. 2010;67(7):835-841. 20625090 10.1001/archneurol.2010.136 PMC3068839

[7] Cherbuin N, Shaw ME, Walsh E, Sachdev P, Anstey KJ. Validated Alzheimer's disease Risk Index (ANU-ADRI) is associated with smaller volumes in the default mode network in the early 60s. Brain Imaging Behav. 2019;13(1):65-74. 29243120 10.1007/s11682-017-9789-5 PMC6409311

[8] Barnes DE, Yaffe K. Predicting dementia: role of dementia risk indices. Future Neurol. 2009;4(5):555-560. 20161571 10.2217/fnl.09.43 PMC2805956

[9] Deckers K, van Boxtel MP, Schiepers OJ, et al. Target risk factors for dementia prevention: a systematic review and Delphi consensus study on the evidence from observational studies. Int J Geriatr Psychiatry. 2015;30(3):234-246. 25504093 10.1002/gps.4245

[10] Schiepers OJG, Köhler S, Deckers K, et al. Lifestyle for Brain Health (LIBRA): a new model for dementia prevention. Int J Geriatr Psychiatry. 2018;33(1):167-175. 28247500 10.1002/gps.4700

[11] Vos SJB, van Boxtel MPJ, Schiepers OJG, et al. Modifiable risk factors for prevention of dementia in midlife, late life and the oldest-old: validation of the LIBRA index. J Alzheimers Dis. 2017;58(2):537-547. 28453475 10.3233/JAD-161208

[12] Pons A, LaMonica HM, Mowszowski L, Köhler S, Deckers K, Naismith SL. Utility of the LIBRA index in relation to cognitive functioning in a clinical health seeking sample. J Alzheimers Dis. 2018;62(1):373-384. 29439337 10.3233/JAD-170731

[13] Deckers K, Nooyens A, van Boxtel M, Verhey F, Verschuren M, Kohler S. Gender and educational differences in the association between lifestyle and cognitive decline over 10 years: the Doetinchem cohort study. J Alzheimers Dis. 2018;70(suppl 1):S31-S41. 10.3233/JAD-180492 PMC6700651 30507570

[14] Deckers K, Barbera M, Köhler S, et al. Long-term dementia risk prediction by the LIBRA score: a 30-year follow-up of the CAIDE study. Int J Geriatr Psychiatry. 2020;35(2):195-203. 31736136 10.1002/gps.5235 PMC7003764

[15] Coley N, Hoevenaar-Blom MP, van Dalen JW, et al. Dementia risk scores as surrogate outcomes for lifestyle-based multidomain prevention trials-rationale, preliminary evidence and challenges. Alzheimers Dement. 2020;16(12):1674-1685 32803862 10.1002/alz.12169

[16] Schram MT, Sep SJ, van der Kallen CJ, et al. The Maastricht study: an extensive phenotyping study on determinants of type 2 diabetes, its complications and its comorbidities. Eur J Epidemiol. 2014;29(6):439-451. 24756374 10.1007/s10654-014-9889-0

[17] Geraets AFJ, Schram MT, Jansen JFA, et al. The relation of depression with structural brain abnormalities and cognitive functioning: The Maastricht study. Psychol Med. 2021;51:1-10. 33634767 10.1017/S0033291721000222 PMC9772903

[18] Deckers K, Kohler S, van Boxtel M, Verhey F, Brayne C, Fleming J. Lack of associations between modifiable risk factors and dementia in the very old: findings from the Cambridge City Over-75s cohort study. Aging Ment Health. 2017;22(10):1272-1278. 28151002 10.1080/13607863.2017.1280767

[19] van Dongen MC, Wijckmans-Duysens NEG, den Biggelaar LJ, et al. The Maastricht FFQ: development and validation of a comprehensive food frequency questionnaire for The Maastricht Study. Nutrition. 2019;62:39-46. 30826598 10.1016/j.nut.2018.10.015

[20] Trichopoulou A, Orfanos P, Norat T, et al. Modified Mediterranean diet and survival: EPIC-Elderly Prospective Cohort study. BMJ. 2005;330(7498):991. 15820966 10.1136/bmj.38415.644155.8F PMC557144

[21] Kromhout D, Spaaij CJ, de Goede J, Weggemans RM. The 2015 Dutch food-based dietary guidelines. Eur J Clin Nutr. 2016;70(8):869-878. 27049034 10.1038/ejcn.2016.52 PMC5399142

[22] Resnicow K, McCarty F, Blissett D, Wang T, Heitzler C, Lee RE. Validity of a modified CHAMPS physical activity questionnaire among African-Americans. Med Sci Sports Exerc. 2003;35(9):1537-1545. 12972874 10.1249/01.MSS.0000084419.64044.2B

[23] Weggemans RM, Backx FJG, Borghouts L, et al. The 2017 Dutch physical activity guidelines. Int J Behav Nutr Phys Act. 2018;15(1):58. 29940977 10.1186/s12966-018-0661-9 PMC6016137

[24] World Health Organization. Obesity. 2019. Accessed February 27, 2019. who.int/topics/obesity/en/.

[25] Sheehan DV, Lecrubier Y, Sheehan KH, et al. The Mini-International Neuropsychiatric Interview (M.I.N.I.): the development and validation of a structured diagnostic psychiatric interview for DSM-IV and ICD-10. J Clin Psychiatry. 1998;59(suppl 20):22-57. 9881538

[26] Kroenke K, Spitzer RL, Williams JB. The PHQ-9: validity of a brief depression severity measure. J Gen Intern Med. 2001;16(9):606-613. 11556941 10.1046/j.1525-1497.2001.016009606.x PMC1495268

[27] Definition and Diagnosis of Diabetes Mellitus and Intermediate Hyperglycaemia: Report of a WHO/IDF Consultation. World Health Organization; 2006.

[28] Rose G, McCartney P, Reid DD. Self-administration of a questionnaire on chest pain and intermittent claudication. Br J Prev Soc Med. 1977;31(1):42-48. 856370 10.1136/jech.31.1.42 PMC478990

[29] Deckers K, Schievink SHJ, Rodriquez MMF, et al. Coronary heart disease and risk for cognitive impairment or dementia: systematic review and meta-analysis. PLoS One. 2017;12(9):e0184244. 28886155 10.1371/journal.pone.0184244 PMC5590905

[30] Inker LA, Schmid CH, Tighiouart H, et al. Estimating glomerular filtration rate from serum creatinine and cystatin C. N Engl J Med. 2012;367(1):20-29. 22762315 10.1056/NEJMoa1114248 PMC4398023

[31] Van der Elst W, van Boxtel MP, van Breukelen GJ, Jolles J. Rey's verbal learning test: normative data for 1855 healthy participants aged 24-81 years and the influence of age, sex, education, and mode of presentation. J Int Neuropsychol Soc. 2005;11(3):290-302. 15892905 10.1017/S1355617705050344

[32] Van der Elst W, Van Boxtel MP, Van Breukelen GJ, Jolles J. The Stroop Color-Word Test: influence of age, sex, and education; and normative data for a large sample across the adult age range. Assessment. 2006;13(1):62-79. 16443719 10.1177/1073191105283427

[33] Van der Elst W, Van Boxtel MP, Van Breukelen GJ, Jolles J. The Concept Shifting Test: adult normative data. Psychol Assess. 2006;18(4):424-432. 17154763 10.1037/1040-3590.18.4.424

[34] van der Elst W, van Boxtel MP, van Breukelen GJ, Jolles J. The Letter Digit Substitution Test: normative data for 1,858 healthy participants aged 24-81 from the Maastricht Aging Study (MAAS): influence of age, education, and sex. J Clin Exp Neuropsychol. 2006;28(6):998-1009. 16822738 10.1080/13803390591004428

[35] Vrooman HA, Cocosco CA, van der Lijn F, et al. Multi-spectral brain tissue segmentation using automatically trained k-nearest-neighbor classification. Neuroimage. 2007;37(1):71-81. 17572111 10.1016/j.neuroimage.2007.05.018

[36] de Boer R, Vrooman HA, van der Lijn F, et al. White matter lesion extension to automatic brain tissue segmentation on MRI. Neuroimage. 2009;45(4):1151-1161. 19344687 10.1016/j.neuroimage.2009.01.011

[37] Fazekas F, Chawluk JB, Alavi A, Hurtig HI, Zimmerman RA. MR signal abnormalities at 1.5 T in Alzheimer's dementia and normal aging. AJR Am J Roentgenol. 1987;149(2):351-356. 3496763 10.2214/ajr.149.2.351

[38] Cordonnier C, Potter GM, Jackson CA, et al. Improving interrater agreement about brain microbleeds: development of the Brain Observer MicroBleed Scale (BOMBS). Stroke. 2009;40(1):94-99. 19008468 10.1161/STROKEAHA.108.526996

[39] Gregoire SM, Chaudhary UJ, Brown MM, et al. The Microbleed Anatomical Rating Scale (MARS): reliability of a tool to map brain microbleeds. Neurology. 2009;73(21):1759-1766. 19933977 10.1212/WNL.0b013e3181c34a7d

[40] Hayes AF, Preacher KJ. Quantifying and testing indirect effects in simple mediation models when the constituent paths are nonlinear. Multivariate Behav Res. 2010;45(4):627-660. 26735713 10.1080/00273171.2010.498290

[41] Lane CA, Hardy J, Schott JM. Alzheimer's disease. Eur J Neurol. 2018;25(1):59-70. 28872215 10.1111/ene.13439

[42] Ferretti MT, Iulita MF, Cavedo E, et al. Sex differences in Alzheimer disease: the gateway to precision medicine. Nat Rev Neurol. 2018;14(8):457-469. 29985474 10.1038/s41582-018-0032-9

[43] Ferretti MT, Martinkova J, Biskup E, et al. Sex and gender differences in Alzheimer's disease: current challenges and implications for clinical practice: position paper of the Dementia and Cognitive Disorders Panel of the European Academy of Neurology. Eur J Neurol. 2020;27(6):928-943. 32056347 10.1111/ene.14174

[44] Armstrong NM, An Y, Beason-Held L, et al. Sex differences in brain aging and predictors of neurodegeneration in cognitively healthy older adults. Neurobiol Aging. 2019;81:146-156. 31280118 10.1016/j.neurobiolaging.2019.05.020 PMC9310670

[45] Berry C, Sidik N, Pereira AC, et al. Small-vessel disease in the heart and brain: current knowledge, unmet therapeutic need, and future directions. J Am Heart Assoc. 2019;8(3):e011104. 30712442 10.1161/JAHA.118.011104 PMC6405580

[46] Qiu C, Winblad B, Fratiglioni L. The age-dependent relation of blood pressure to cognitive function and dementia. Lancet Neurol. 2005;4(8):487-499. 16033691 10.1016/S1474-4422(05)70141-1

[47] O'Brien JT, Markus HS. Vascular risk factors and Alzheimer's disease. BMC Med. 2014;12(1):218. 25385509 10.1186/s12916-014-0218-y PMC4226870

[48] Bos I, Vos SJB, Schindler SE, et al. Vascular risk factors are associated with longitudinal changes in cerebrospinal fluid tau markers and cognition in preclinical Alzheimer's disease. Alzheimers Dement. 2019;15(9):1149-1159. 31378575 10.1016/j.jalz.2019.04.015 PMC6756978

[49] Salvadó G, Brugulat-Serrat A, Sudre CH, et al. Spatial patterns of white matter hyperintensities associated with Alzheimer's disease risk factors in a cognitively healthy middle-aged cohort. Alzheimers Res Ther. 2019;11(1):12. 30678723 10.1186/s13195-018-0460-1 PMC6346579

[50] Licher S, Yilmaz P, Leening MJG, et al. External validation of four dementia prediction models for use in the general community-dwelling population: a comparative analysis from the Rotterdam Study. Eur J Epidemiol. 2018;33(7):645-655. 29740780 10.1007/s10654-018-0403-y PMC6061119

